# Hinokitiol Negatively Regulates Immune Responses through Cell Cycle Arrest in Concanavalin A-Activated Lymphocytes

**DOI:** 10.1155/2015/595824

**Published:** 2015-08-26

**Authors:** Chi-Li Chung, Kam-Wing Leung, Wan-Jung Lu, Ting-Lin Yen, Chia-Fu He, Joen-Rong Sheu, Kuan-Hung Lin, Li-Ming Lien

**Affiliations:** ^1^Division of Pulmonary Medicine, Department of Internal Medicine, Taipei Medical University Hospital, Taipei 110, Taiwan; ^2^School of Respiratory Therapy, College of Medicine, Taipei Medical University, Taipei 110, Taiwan; ^3^Department of Dentistry, Yuan's General Hospital, Kaohsiung 802, Taiwan; ^4^Department of Pharmacology and Graduate Institute of Medical Sciences, College of Medicine, Taipei Medical University, Taipei 110, Taiwan; ^5^Central Laboratory, Shin Kong Wu Ho-Su Memorial Hospital, Taipei 111, Taiwan; ^6^School of Medicine, College of Medicine, Taipei Medical University, Taipei 110, Taiwan; ^7^Department of Neurology, Shin Kong Wu Ho-Su Memorial Hospital, Taipei 111, Taiwan

## Abstract

Autoimmune diseases are a group of chronic inflammatory diseases that arise from inappropriate inflammatory responses. Hinokitiol, isolated from the wood of *Chamaecyparis taiwanensis*, engages in multiple biological activities. Although hinokitiol has been reported to inhibit inflammation, its immunological regulation in lymphocytes remains incomplete. Thus, we determined the effects of hinokitiol on concanavalin A- (ConA-) stimulated T lymphocytes from the spleens of mice. In the present study, the MTT assay revealed that hinokitiol (1–5 *μ*M) alone did not affect cell viability of lymphocytes, but at the concentration of 5 *μ*M it could reduce ConA-stimulated T lymphocyte proliferation. Moreover, propidium iodide (PI) staining revealed that hinokitiol arrested cell cycle of T lymphocytes at the G0/G1 phase. Hinokitiol also reduced interferon gamma (IFN-*γ*) secretion from ConA-activated T lymphocytes, as detected by an ELISA assay. In addition, hinokitiol also downregulated cyclin D3, E2F1, and Cdk4 expression and upregulated p21 expression. These results revealed that hinokitiol may regulate immune responses. In conclusion, we for the first time demonstrated that hinokitiol upregulates p21 expression and attenuates IFN-*γ* secretion in ConA-stimulated T lymphocytes, thereby arresting cell cycle at the G0/G1 phase. In addition, our findings also indicated that hinokitiol may provide benefits to treating patients with autoimmune diseases.

## 1. Introduction

Mature lymphocytes must proliferate intensely and repeatedly to provide a rapid immune response and generate immunological memory [1]. Cell proliferation is a mandatory process for immune-system function. However, unregulated or excessive immune responses may cause immune-mediated inflammatory diseases (IMIDs) such as rheumatoid arthritis, Crohn's disease, systemic lupus erythematosus (SLE), and multiple sclerosis [2, 3]. These diseases are commonly T lymphocyte-mediated disorders. Although the pathogenic mechanisms underlying the development of these diseases are not entirely clear, studies have proposed that increased lymphocyte cycling or defective apoptosis may cause breakdown of immune tolerance and autoimmunity as well as lymphoma generation [1–3]. Thus, controlling the cell cycle of lymphocytes may be an effective therapeutic strategy for treating patients with IMIDs.

The cell cycle inhibitor p21, which belongs to the Cip/Kip family, interferes with cycling by inhibiting all cyclin-dependent kinases (CDKs) involved in the G1/S phase, thereby controlling cell proliferation and tumorigenesis in various cell types [4]. In addition, p21 deficiency was reported to enhance T lymphocyte activation and proliferation and to induce autoimmune manifestations [5]. Suppression of p21 promotes malignant T lymphocyte proliferation in malignant CD30^+^ T lymphocytes [6]. Thus, p21 may play a critical role in autoimmune diseases and tumorigenesis by regulating T lymphocyte activation and proliferation.

Hinokitiol is a naturally occurring compound isolated from the wood of* Chamaecyparis taiwanensis* [7]. Hinokitiol has been used in hair tonics, tooth pastes, cosmetics, and food as an antimicrobial agent [8]. Moreover, hinokitiol engages in multiple biological activities, including anticancer and anti-inflammatory activities [9, 10]. Studies have reported that hinokitiol suppresses tumor growth by inhibiting cell proliferation and inducing apoptosis or autophagy in various cancer cell lines [9, 11–13]. It was also reported to suppress tumor necrosis factor *α* production by inhibiting NF-*κ*B activity in lipopolysaccharide-stimulated macrophages [10]. In our previous study, we demonstrated that hinokitiol exhibits potent antiplatelet activity [14].

Although hinokitiol has been reported to engage in multiple biological activities, the regulation of lymphocytes by hinokitiol has not been fully investigated. In our preliminary study, we determined that hinokitiol can arrest the cell cycle of T lymphocytes. Thus, we evaluated the effects of hinokitiol in concanavalin A- (ConA-) activated T lymphocytes isolated from the spleens of mice.

## 2. Materials and Methods

### 2.1. Materials

Hinokitiol was purchased from Sigma (St. Louis, MO). The anticyclin D3, anti-E2F1, anti-Cdk4, and anti-GAPDH polyclonal antibodies (pAbs) and anti-p21 monoclonal antibody (mAb) were purchased from GeneTex (Irvine, CA). The PI-annexin V-FITC kit was purchased from BioLegend (San Diego, CA). The Mouse Interferon Gamma (IFN-*γ*) ELISA Ready-SET-Go kit was purchased from eBioscience (San Diego, CA). The Hybond-P polyvinylidene difluoride membrane, an enhanced chemiluminescence (ECL) western blotting detection reagent and analysis system, the horseradish peroxidase- (HRP-) conjugated donkey anti-rabbit immunoglobulin G (IgG), and the sheep anti-mouse IgG were purchased from Amersham (Buckinghamshire, UK). Hinokitiol was dissolved in 0.5% dimethyl sulfoxide (DMSO) and stored at 4°C until used.

### 2.2. Mice

The protocols conformed to the Guide for the Care and Use of Laboratory Animals (NIH publication number 85–23, 1996). Briefly, male BALB/c mice (6–8 weeks old, approximately 20–25 g) were purchased from BioLASCO Taiwan Co. Ltd. and fed in the animal house of Taipei Medical University.

### 2.3. Lymphocyte Preparation

The spleen was aseptically removed from each mouse and placed in a sterile petri dish containing the RPMI 1640 medium. Single-cell suspensions were prepared by gently disrupting the spleen on a sterile wire mesh. The cell suspensions were centrifuged at 300 g for 5 min, and red blood cells were then lysed using the ACK (ammonium-chloride-potassium) lysis buffer (15 mL) and, subsequently, 1x phosphate buffered saline (PBS; 20 mL). The lymphocyte pellets were collected through centrifugation at 300 g for 5 min and suspended with RPMI containing 5% heat-inactivated fetal bovine serum (Gibco). The cell viability was determined according to trypan blue exclusion. The cells were prepared at an appropriate density depending on the scale of each experiment.

### 2.4. Cell Viability

Cell proliferation was evaluated using a colorimetric assay. Cell viability was measured by conducting a 3-(4,5-dimethylthiazol-2-yl)-2,5-diphenyl tetrazolium bromide (MTT) assay. In brief, cells (3 × 10^5^ cells/well) were cultured in 96-well plates and incubated with a vehicle or hinokitiol (1, 2, or 5 *μ*M) for 24 or 48 h. MTT (5 mg/mL) was added and the cells were incubated for an additional 1 h. The cells were then lysed in 400 *μ*L of DMSO. The absorbance was measured at 570 nm by using a microplate reader. Each experiment was performed in triplicate and repeated at least three times.

### 2.5. Cytokine Secretion according to ELISA Assay

The amounts of secreted IFN-*γ* protein were quantified using the Mouse IFN-*γ* ELISA Ready-SET-Go kit (eBioscience, San Diego, CA). Recombinant IFN-*γ* was used to generate a standard curve, which was employed in calculating the IFN-*γ* concentrations of all samples. All procedures were performed according to the manufacturer's instructions (eBioscience).

### 2.6. Flow Cytometric Analysis

Cells were cultured in 24-well plates. After reaching 80% confluence, the cells were treated with a vehicle or hinokitiol (1, 2, or 5 *μ*M) for 48 h. The cells were washed twice with PBS, detached, and centrifuged. The cells (1 × 10^6^) were then resuspended with 0.5 mL of PBS and then added to propidium iodide (PI, 50 *μ*g/mL) for 15 min at room temperature in the dark before flow cytometric analysis was conducted. Finally, the cells were filtered on a nylon mesh filter. The samples were analyzed using a flow cytometer (Becton Dickinson, FACScan Syst., San Jose, CA). Each experiment was repeated at least three times.

### 2.7. Immunoblotting

Cells (1 × 10^7^) were cultured in 6-well plates. After reaching 80% confluence, the cells were treated with a vehicle or hinokitiol (1, 2, or 5 *μ*M) for 24 h. After the reactions, the cells were collected and lysed with 70 *μ*L of a lysis buffer. Samples containing 40 *μ*g of protein were separated by conducting sodium dodecyl sulfate polyacrylamide gel electrophoresis. The proteins were electrotransferred by a Bio-Rad semidry transfer (Hercules, CA). The membranes were blocked with TBST (10 mM Tris-base, 100 mM NaCl, and 0.01% Tween 20) containing 5% BSA for 1 h and then probed with various primary antibodies. Membranes were incubated with the HRP-linked anti-mouse IgG or anti-rabbit IgG (diluted 1 : 3000 in TBST) for 1 h. Immunoreactive bands were detected using an ECL system. Semiquantitative results were obtained by scanning reactive bands and quantifying the optical density of each band by using videodensitometry (Bio-profil; Biolight Windows Application V2000.01; Vilber Lourmat, France).

### 2.8. Data Analysis

The experimental results are expressed as the mean ± SEM and are accompanied by the number of observations. The data were assessed by conducting an analysis of variance. When this analysis indicated significant differences among the group means, further comparisons were made using the Newman-Keuls method. *P* < 0.05 indicated statistical significance.

## 3. Results

### 3.1. Hinokitiol Reduces the Viability and Cytokine Secretion of Lymphocytes

**Figure 1:**
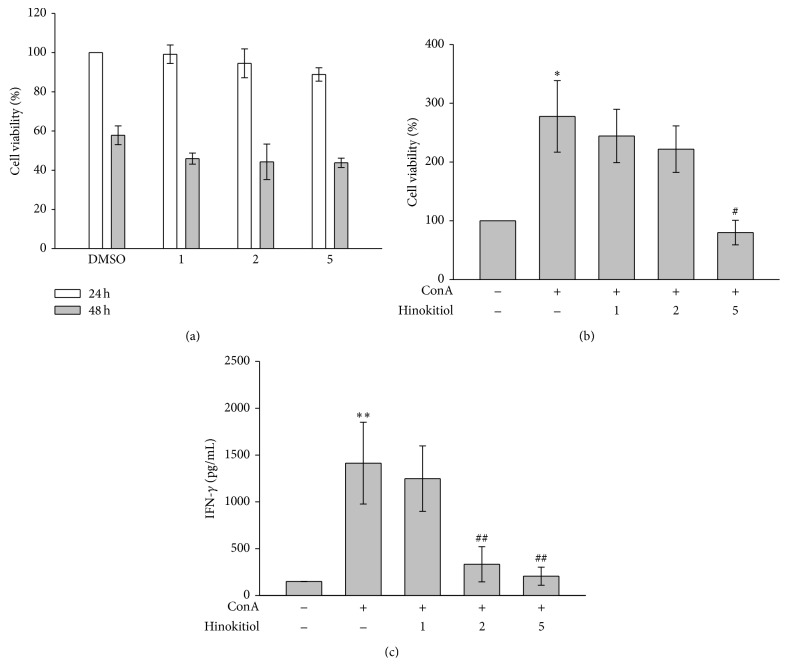
Effects of hinokitiol on cell viability and interferon gamma (IFN-*γ*) secretion in ConA-activated T lymphocytes. Cells were treated with hinokitiol (1–5 *μ*M) in the absence or presence of ConA (10 *μ*g/mL) for 24 or 48 h. (a, b) Cell viability was determined using a MTT assay (*n* = 4). (c) The level of IFN-*γ* was measured by an ELISA assay (*n* = 3). Data (b, c) are presented as the mean ± SEM (^*^ *P* < 0.05 and ^**^ *P* < 0.01 compared with solvent control (DMSO); ^#^ *P* < 0.05 and ^##^ *P* < 0.01 compared with the ConA-treated group).

In the present study, an MTT assay was used to evaluate the cell viability and proliferation of lymphocytes. As shown in Figure 1(a), hinokitiol at the concentrations of 1, 2, and 5 *μ*M did not affect the viability of lymphocytes after treatment for 24 and 48 h, indicating that hinokitiol (≤5 *μ*M) did not exhibit cytotoxicity to lymphocytes.  Figure 1(b) shows that ConA treatment (10 *μ*g/mL) for 24 h induced lymphocyte proliferation, which was reversed by 5 *μ*M hinokitiol, indicating that hinokitiol inhibits ConA-induced cell proliferation of lymphocytes. In addition, we determined the influence of hinokitiol on the levels of IFN-*γ* secreted from ConA-stimulated T lymphocytes (Figure 1(c)).

### 3.2. Hinokitiol Arrests the Cell Cycle at the G0/G1 Phase

**Figure 2:**
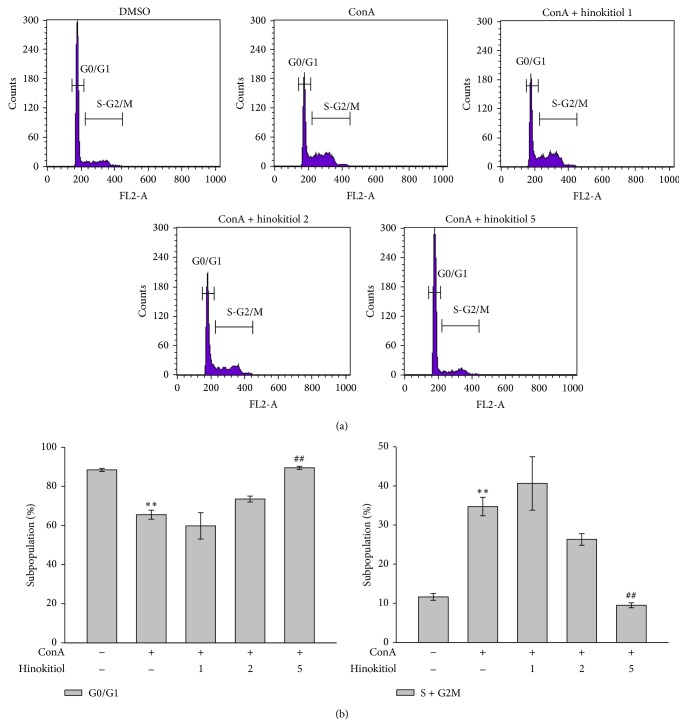
Effects of hinokitiol on the cell cycle in ConA-activated T lymphocytes. Cells were treated with hinokitiol (1–5 *μ*M) in the absence or presence of ConA (10 *μ*g/mL) for 48 h. (a) Cell cycle was determined by PI staining under a flow cytometry. (b) The panel shows the population of the G0/G1 and S-G2/M phases. Data (b) are presented as the mean ± SEM (*n* = 3; ^**^ *P* < 0.01 compared with solvent control (DMSO); ^##^ *P* < 0.01 compared with the ConA-treated group).

PI staining was used to determine the effect of hinokitiol on the cell cycle in ConA-activated lymphocytes. Following ConA stimulation for 48 h, quiescent lymphocytes (G0) began cycling. The population of the G0/G1 phase decreased 22.9% and the population of the S and G2/M phases increased 23.1% upon ConA treatment compared with nontreatment (resting); these changes were reversed by 5 *μ*M hinokitiol (Figures 2(a) and 2(b)). Hinokitiol markedly arrested the cell cycle at the G0/G1 phase in ConA-stimulated lymphocytes (Figure 2(a)). Compared with ConA treatment, 5 *μ*M hinokitiol treatment increased the population of the G0/G1 phase by 24% and reduced the population of the S and G2/M phases by 25.2% (Figures 2(a) and 2(b)).

### 3.3. Hinokitiol Downregulates the Expression of the Cyclin D3, Cdk4, and E2F1 Proteins and Upregulates the Expression of the p21 Protein

**Figure 3:**
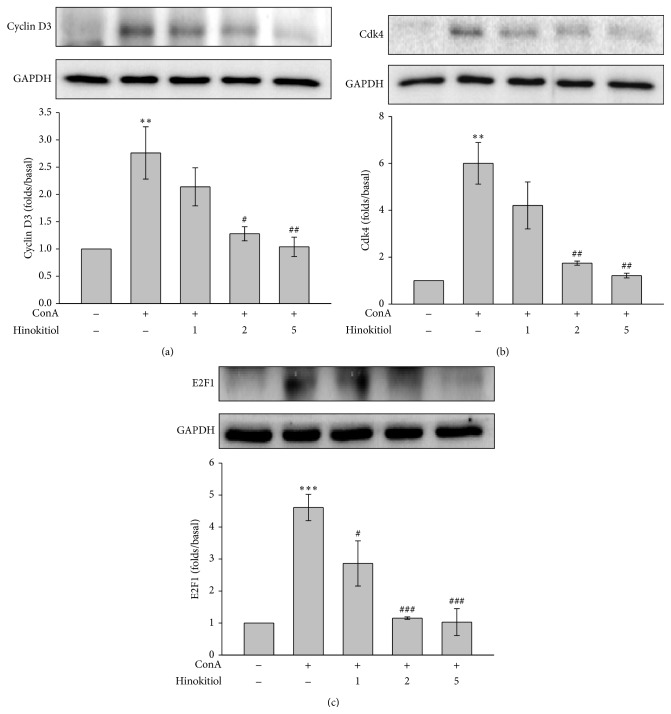
Effects of hinokitiol on positive regulators of the cell cycle. Cells were treated with hinokitiol (1–5 *μ*M) in the absence or presence of ConA (10 *μ*g/mL) for 24 h. The specific antibodies were used to detect (a) cyclin D3, (b) Cdk4, and (c) E2F1. Data (a–c) are presented as the mean ± SEM (*n* = 3; ^**^ *P* < 0.01 and ^***^ *P* < 0.001 compared with solvent control (DMSO); ^#^ *P* < 0.05, ^##^ *P* < 0.01, and ^###^ *P* < 0.001 compared with the ConA-treated group).

**Figure 4:**
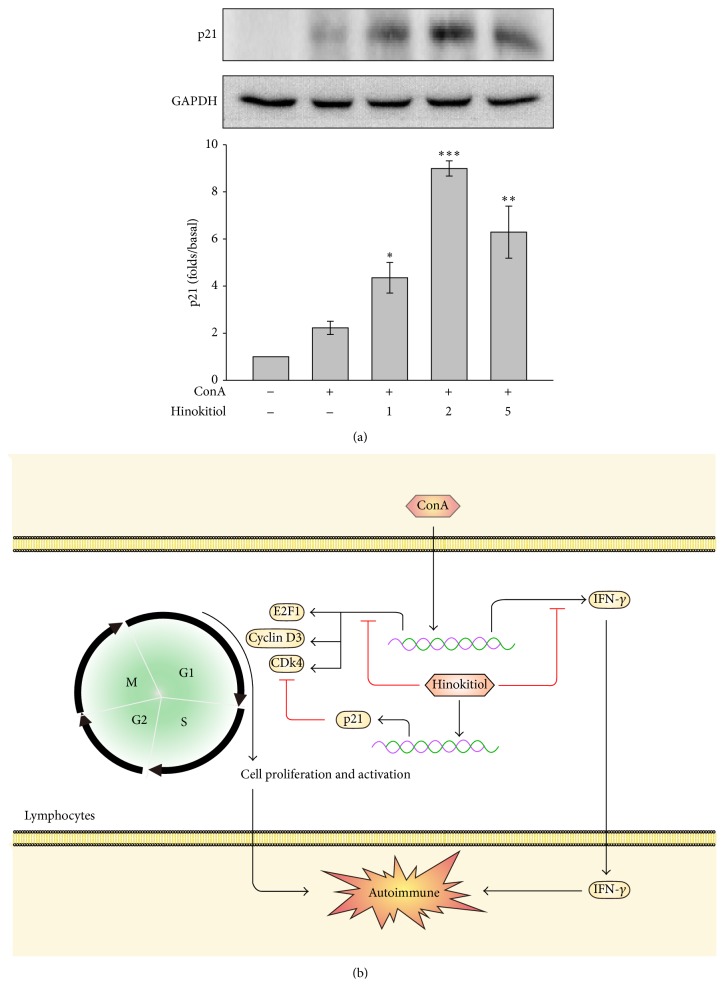
Effects of hinokitiol on negative regulators of the cell cycle. (a) Cells were treated with hinokitiol (1–5 *μ*M) in the presence of ConA (10 *μ*g/mL) for 24 h. The specific antibody was used to detect p21. Data are presented as the mean ± SEM (*n* = 3; ^*^ *P* < 0.05, ^**^ *P* < 0.01, and ^***^ *P* < 0.001 compared with the ConA (alone)-treated group). (b) Schematic illustration of hinokitiol-mediated inhibition of immune responses in ConA-activated T lymphocytes. Hinokitiol downregulates cyclin D3, Cdk4, and E2F1 expression and upregulates p21 expression and subsequently arrests the cell cycle at the G0/G1 phase. Hinokitiol also attenuates IFN-*γ* secretion. Finally, hinokitiol negatively regulates immune responses.

The processes of cell cycling are complex and involve positive regulators such as cyclin D3, Cdk4, and E2F1 and negative regulators such as p21. These proteins were determined in this study. Our data revealed that 5 *μ*M hinokitiol significantly inhibited ConA-induced cyclin D3 and Cdk4 expression (Figures 3(a) and 3(b)) and downregulated the transcriptional factor E2F1 (Figure 3(c)). In addition, hinokitiol upregulated the cell cycle inhibitor p21 (Figure 4(a)).

## 4. Discussion

In the present study, we, for the first time, demonstrated that hinokitiol negatively regulates immune responses by arresting the G0/G1 phase of the cell cycle in ConA-activated T lymphocytes. Hinokitiol, a tropolone-related compound found in heartwood cupressaceous plants, exhibits multiple biological activities, including anti-inflammatory, antitumorigenic, and antiplatelet activities [9, 10, 14]. However, the regulation of lymphocytes by hinokitiol has not been fully investigated. Thus, in the present study, we examined the mechanisms underlying the regulation of T lymphocytes by hinokitiol. The lectin ConA from the jack bean (*Canavalia ensiformis*) has been used widely as a T lymphocytes-specific mitogen and to induce the proliferation of lymphocytes [15]. Thus, we used this model to investigate the effect of hinokitiol on T lymphocytes in response to ConA.

Dysregulation of the immune system may lead to various chronic diseases such as autoimmune diseases. Most of the damage inflicted by autoimmune diseases is the result of inappropriate inflammatory responses [16]. Failure of self-tolerance is the fundamental cause of autoimmunity. The principal mechanisms of peripheral tolerance are anergy (functional unresponsiveness), deletion (apoptotic cell death), and suppression by regulatory T cells [17]. A previous study reported that increased cell cycling or defective apoptosis of lymphocytes may lead to a break of tolerance and autoimmunity [1]. The cell cycle is a complex process that involves positive regulators such as cyclins and CDKs and negative regulators such as CDK inhibitors. CDK inhibitors are classified into two families, INK4 and Cip/Kip. During the G1-S transition, cyclins (D2 and D3) and CDKs (4 and 6) are upregulated. By contrast, the cell cycle inhibitor p21, which belongs to the Cip/Kip family, interferes with cycling by inhibiting all CDKs involved in the G1/S phase [1].

In the present study, we observed that hinokitiol arrested the cell cycle of T lymphocytes by suppressing cyclin D3, Cdk4, and E2F1 expression and upregulating p21 expression. A study reported that p21 controls T lymphocyte proliferation [18], and Trivedi et al. indicated that NK cells inhibit T lymphocyte proliferation by upregulating p21, resulting in cell cycle arrest at the G0/G1 phase [19]. The findings of these studies are consistent with our findings that p21 upregulation by hinokitiol leads to G0/G1 arrest. In addition to negatively regulating the cell cycle, p21 was reported be associated with tolerance and systemic autoimmune disease. Loss of tolerance was observed in p21^−/−^ mice, of which the T lymphocytes became more proliferative in response to stimulation. These mice also exhibited an SLE-like syndrome characterized by the development of anti-DNA antibodies and glomerulonephritis [18, 20]. These observations suggest that hinokitiol prevents autoimmune responses by upregulating p21.

In addition, IFN-*γ* is crucial for immunity to pathogens. IFN-*γ* is mainly produced in T lymphocytes, NKT cells, NK cells, and B cells [21]. T lymphocytes are the major sources of IFN-*γ* in adaptive immune responses [21]. Studies have reported that increased IFN-*γ* production is associated with greater antibacterial and antiviral effects [22, 23]. However, aberrant IFN-*γ* expression has been associated with inflammatory diseases. Jaruga et al. demonstrated that IFN-*γ* plays a vital role in ConA-activated T cell hepatitis by enabling leucocytes to infiltrate the liver [24]. Moreover, excess IFN-*γ* has been associated with chronic autoimmune diseases, including inflammatory bowel disease, multiple sclerosis, diabetes mellitus, and SLE [25, 26]. Thus, we determined the effect of hinokitiol on IFN-*γ* expression in ConA-stimulated T lymphocytes and observed that hinokitiol significantly prevented IFN-*γ* expression.

In clinical practice, therapies for autoimmune diseases primarily involve using powerful agents, chemicals, or biologics (corticosteroids, thiopurines, methotrexate, cyclosporine, and antitumor necrosis factor agents) [27]. Such agents suppress the global immune system but frequently cause undesirable side effects. Certain studies have reported that immunosuppressive drugs can increase the risk of cancer and infectious complications [28–31]. Regarding this part, we demonstrated that hinokitiol exerts immunosuppressive effects. Moreover, previous studies have proved that hinokitiol engages in antitumor and antibacterial activities. Whether these beneficial effects of hinokitiol reduce the incidence of side effects associated with immune suppression warrants investigation.

In summary, we observed that hinokitiol inhibits the activation and proliferation of T lymphocytes by arresting the cell cycle at the G0/G1 phase, upregulating p21 expression, and preventing IFN-*γ* production (Figure 4(b)). Because it engages in multiple biological activities, especially anti-inflammatory and antitumorigenic activities, hinokitiol may reduce the unexpected occurrence of side effects during the treatment of patients with autoimmune diseases. Thus, the results of our study suggest that hinokitiol provides benefits in treating autoimmune diseases.
